# Short- and long-term outcomes after colonic self-expandable metal stent placement for malignant large-bowel obstruction as a bridge to surgery focus on the feasibility of the laparoscopic approach: a retrospective, single center study

**DOI:** 10.1186/s12957-020-02039-8

**Published:** 2020-10-13

**Authors:** Jesse Yu Tajima, Nobuhisa Matsuhashi, Takao Takahashi, Chika Mizutani, Yoshinori Iwata, Shigeru Kiyama, Masaya Kubota, Takashi Ibuka, Hiroshi Araki, Masahito Shimizu, Kiyoshi Doi, Kazuhiro Yoshida

**Affiliations:** 1 Department of Surgical Oncology, Gifu University Hospital, 1-1 Yanagido, Gifu-city, Gifu, 501-1194 Japan; 2 Department of Gastroenterology, Gifu University Hospital, Gifu, Japan; 3 General and Cardiothoracic Surgery, Gifu University Hospital, Gifu, Japan

**Keywords:** Malignant large-bowel obstruction, Self-expandable metal stent placement, Bridge to surgery, Postoperative complication

## Abstract

**Purpose:**

Malignant large-bowel obstruction (MLBO) is a highly urgent condition in colorectal cancer with high complication rates. Self-expandable metal stent (SEMS) placement in MLBO is a new decompression treatment in Japan. Preoperative stent placement (bridge to surgery: BTS) avoids emergency surgery, but oncological influences of stent placement and post-BTS surgical approach remain unclear. We examined short- and long-term results of surgery for MLBO after SEMS placement in our hospital.

**Methods:**

We retrospectively reviewed 75 patients with MLBO who underwent resection after SEMS placement at our hospital from June 2013 to December 2018. Postoperative morbidity and mortality were evaluated by comparison with the surgical approach.

**Results:**

Tumor location was significantly higher in the left-side colon and rectum (*n* = 59, 78.7%) than right-side colon (*n* = 16, 21.3%). Technical and clinical success rates for SEMS placement were 97.3% and 96.0%, respectively. Laparoscopic surgery was performed in 54 patients (69.0%), and one-stage anastomosis was performed in 73 (97.3%). Postoperative complications were similar in the open surgery (open) group (*n* = 5, 23.8%) and laparoscopic surgery (lap) group (*n* = 7, 13.0%), with no severe complications requiring reoperation. Three-year overall survival (OS) and relapse-free survival (RFS) rates were not significantly different in the lap vs open group (67.5% vs 66.4%; 82.2% vs 62.5%).

**Conclusion:**

Preoperative stent treatment avoids stoma construction but allows anastomosis. One-time surgery was performed safely contributing to minimally invasive treatment and acceptable short- and long-term results.

## Introduction

Colorectal cancer is the fourth most common malignant disease and the fifth most frequent cause of cancer-related deaths worldwide [1]. With the recent increase in colorectal cancer patients, the incidence of malignant large-bowel obstruction (MLBO) has also been increasing. MLBO is reported in 3–29% of all colorectal cancers, and thus is not a rare condition but one that is frequently encountered clinically [2–6]. Most cases of MLBO occur in the left-side colon because of solid stool [7]. MLBO is a highly urgent condition [6], and the complication rate following emergency surgery ranges as high as 30–60% [8]. Even if anastomosis is performed simultaneously with resection, construction of a colostomy is inevitable in many patients because of the high rate of postoperative complications, especially anastomotic leakage. Among them, many patients eventually progress to a permanent stoma, which greatly lowers quality of life. From this background, transanal drainage or colonic stent placement has begun to be performed to avoid emergency surgery [9–11].

A colonic stent was first inserted as a palliative treatment by Dohmoto in 1991 using an esophageal stent [12]. Since then it has been widely used for both palliative care and as a bridge to surgery (BTS), mainly in western countries. In Japan, since implantation of an indwelling self-expanding metallic stent (SEMS) for MLBO began to be covered by insurance in January 2012, the number of SEMS placements has increased significantly. At the same time as the insurance indication was granted, a study group for determining safe techniques of colonic stent implantation was launched, which reported the safety and effectiveness of colonic stenting through multicenter prospective research [13, 14]. Recently, the European Society of Gastrointestinal Endoscopy (ESGE) announced clinical guidelines for SEMS placement for MLBO in 2020 [15]. The ESGE recommended SEMS as a BTS as a treatment for curable MLBO within the condition of decision-making process by experts. This recommendation is based on several analyzes that stent-related perforations increase the risk of recurrence and even influence the long-term outcome [16].

At present, improvements in technique and maintenance of guidelines have resulted in a > 90% success rate for colonic stent placement, which has contributed to increasing the number of institutions actively adopting BTS [17].

However, many points regarding the influence on tumors of stent placement and on the long-term results remain unclear. The surgical approach after SEMS placement is also another problem. Results of a questionnaire survey in Japan showed that laparoscopic surgery has been performed in 70.1% of all colorectal cancers, and the rate continues to increase rapidly [18]. However, there are several concerns about the laparoscopic approach in BTS cases such as oncological curability, difficulty of the technique, and other issues.

To solve these problems, we examined BTS cases in which SEMS was placed at our institution.

## Patients and methods

### Patients

We retrospectively reviewed patients with MLBO who underwent resection after SEMS placement at our hospital from June 2013 to December 2018. In addition to clinical symptoms which is defined as less than score2 with ColoRectal Obstruction Scoring System (CROSS) such as abdominal pain, abdominal distention, nausea, vomiting, or constipation, the dilation of the small or large bowel on computed tomography (CT) or a stenosis that could not be passed by an endoscope was selected as indication of stent placement [19]. A histopathological diagnosis was made following endoscopic biopsy in all cases, and the tumor was staged according to the UICC 8th edition. This study was approved by the central ethics committee of Gifu University (approved number: 2019-195).

### Endoscopic stenting procedure and follow-up of BTS

All SEMS placements were performed by well-experienced endoscopists using a WallFlex colonic stent (Boston Scientific, Marlborough, MA) or Niti-S colonic stent (Taewoong Medical Inc., Gimpo-si, Korea). WallFlex colonic stent was used in the first 5 cases, and Niti-S colonic stent was used thereafter cases. Stent size (18 or 22 mm in diameter, 60–120 mm in length) was selected by the endoscopists according to the size and location of the tumor. The treatment was selected based on the patient’s own decision after the informed consent of the benefits and the risks of insertion of SEMS compared with emergency surgery. We defined the technical success rate as the rate of patients in whom the stent could be inserted properly and the clinical success rate as the rate of patients in whom technical success was achieved and the obstruction was released without complications until surgery could be performed.

After SEMS insertion, improvement of the obstruction was monitored by the patients’ abdominal symptoms and by abdominal X-ray. If improvement of the obstruction could be confirmed, water intake was started from the day after SEMS placement, and oral intake was started from the third day with magnesium-oxide agents after the placement. One week after stent placement, a colonoscopy was performed again to examine the oral side of the tumor. The cases of indication for endoscopic resection (ER), the ER were performed and the necessity of additional resection was determine by a pathological evaluation before the surgery.

### Clavien-Dindo classification

We evaluated the safety and feasibility of the procedure using the Clavien-Dindo classification, which categorizes surgical complications from grades I to V based on the invasiveness of necessary treatment [20]. Grade I requires no treatment or wound infection opened at the bedside; grade II requires medical therapy; grade IIIa requires surgical, endoscopic, or radiological intervention but not general anesthesia, and grade IIIb requires general anesthesia; grade IV represents life-threatening complications that require intensive care; and grade V represents patient death. We retrospectively reviewed the patients’ records to determine the incidence of complications of grades II to V during hospitalization and within 30 days after surgery. With the exception of surgical site infections, we did not evaluate grade I complications so as to exclude the possibility of description bias in the patient records. Serious complications were defined as complications of grade IIIa or higher. Mortality (grade V) was defined as hospital death due to any cause after surgery.

### Statistical analysis

Statistical analysis was carried out using the JMP® 14.1 software (SAS Institute Inc., Cary, NC, USA). Multivariate *p* values were used to characterize the independence of the factors. A 95% confidence interval (CI) was used to quantify the relationship between survival time and each independent factor. All *p* values were 2 sided in the tests, and *p* values ≤ 0.05 were considered significant. Survival analysis and curves were established according to the Kaplan-Meier method and compared using the log­rank test. Median follow­up time was calculated as the median observation interval for all patients, being the time from diagnosis or colorectal stenting for obstruction to the last follow­up or death. Relapse­free survival (RFS) was defined as the time since diagnosis or stenting to the first evidence of relapse in stage I–III patients. Additionally, overall survival (OS) was defined as the time from diagnosis to the date of the patients’ death or last known contact.

## Results

### Patients characteristics

We examined 75 patients with MLBO whose background is shown in Table 1. The median age was 66 years old (range, 30–86 years). The male-female ratio was 48 men (64.0%) to 27 women (36.0%). In staging based on the UICC, there was 1 patient with stage I, 26 (34.7%) patients with stage II, 26 (34.7%) with stage III, and 22 (29.3%) with stage IV. Tumor location was significantly higher in the left-side colon and rectum (*n* = 59, 78.7%) than in the right-side colon (*n* = 16, 21.3%).

**Table 1:** Characteristics of the patients

Characteristics			*N* = 75
Age (year)*			66 (30-86)
Sex (%)		Male	48 (64.0)
		Female	27 (36.0)
BMI*			21.1 (14.4-34.9)
ASA (%)		1	25 (33.3)
		2-3	50 (66.7)
Tumor location (%)	Right	A	6 (8.0)
		T	10 (13.3)
	Left	D	7 (9.3)
		S	28 (37.3)
		RS	17 (22.7)
		Ra	6 (8.0)
		Rb	1 (1.3)
T factor (%)		2	1 (1.3)
		3	40 (53.3)
		4a	26 (34.7)
		4b	8 (10.7)
N factor (%)		0	30 (40.0)
		1	23 (30.7)
		2	22 (29.3)
TNM stage (%)		I	1 (1.3)
		IIA/IIB/IIC	10/12/4 (34.7)
		IIIA/IIIB/IIIC	0/18/8 (34.7)
		IVA/IVB	21/1 (29.3)
TSR (%)			73/75 (97.3)
CSR (%)			72/75 (96.0)
Interval to surgery (day)*			13 (0-62)
Synchronous cancers (%)			11 (14.7)

*BMI* body mass index, *ASA* American Society of Anesthesiologists, Prognostic Score, *A* ascending colon, *T* transverse colon, *D* descending colon, *S* sigmoid colon, *RS* rectosigmoid colon, *Ra* rectum above the peritoneal reflection, *Rb* rectum below the peritoneal reflection, *TSR* technical success rate, *CSR* clinical success rate

*Median (min-max)

The technical success rate of SEMS placement was 97.3%, and the clinical success rate was 96.0%. Two patients were confirmed to have suffered perforation during the procedure (perforation rate, 2.7%), and emergency surgery was performed on the same day. Stenosis of the SEMS occurred in one patient during the interval to surgery. Staging CT scans was given in all of above 3 cases before inserting SEMS and no synchronous malignancy was found. Except for these 3 patients, the remaining 72 patients showed improvement of obstructive symptoms, it was possible to perform total colonoscopy and elective surgery without shifting to an emergency surgery during the follow-up period. Additionally, during BTS follow-up, synchronous multiple cancers were detected in 11 of the 75 patients (14.7%), of whom 4 required a change in procedure (Table 2). Table 3 shows the operative background and about the presence of adjuvant chemotherapy and recurrence. The median time from SEMS placement to surgery was 13 days (range, 0–62 days). A one-step anastomosis was performed in 73 patients (97.3%). The rate of Clavien-Dindo grade II or higher complications was 28.0% (*n* = 21) and that of grade III or higher was 16.0% (*n* = 12). Although 7 patients suffered anastomotic leakage, all improved with conservative treatment, and none required reoperation. Among 52 patients who underwent curative resection, 37 patients (71.2%) received adjuvant chemotherapy and 7 (13.5%) had recurrences. The sites of the recurrence were 1 liver metastasis, 2 peritoneal dissemination, 1 lung metastasis, 1 brain metastasis, and 3 lymph node metastases (para-aortic or lateral pelvic).

**Table 2:** Synchronous cancers among the MLBO patients

No.	Obstructed site	Synchronous cancers site	Change of surgical procedure	Approach	Operation
1	S	D	No	Open	Anterior resection
2	RS	C	Yes	Lap	Anterior resection, right hemicolectomy
3	S	Rb	No	Open	Very low anterior resection, covering colostomy
4	S	Rb	No	Open	Abdominoperineal resection
5	Ra	S, FAP	Yes	Lap	Total colectomy
6	RS	T, gastric cancer	Yes	Lap	Low anterior resection, partial transverse colectomy, distal gastrectomy
7	A	S (ESD)	No	Lap	Sigmoidectomy
8	S	Renal pelvic cancer	Yes	Open	Sigmoidectomy, right nephrectomy
9	S	T (ESD)	No	Lap	Sigmoidectomy
10	D	S	No	Lap	Left hemicolectomy
11	T	S	No	Lap	Left hemicolectomy

*C* cecum, *A* ascending colon, *T* transverse colon, *D* descending colon, *S* sigmoid colon, *RS* rectosigmoid colon, *Ra* rectum above the peritoneal reflection, *Rb* rectum below the peritoneal reflection, *FAP* familial adenomatous polyposis, *ESD* endoscopic submucosal dissection

**Table 3:** Perioperative features

Characteristics		*N* = 75
Period to surgery (day)*		13 (0-62)
Approach of surgery (%)	Open	21 (28.0)
	Lap	54 (72.0)
Tumor size (mm)*		65 (40-112)
Lymph node dissection*		25 (7-102)
Blood loss (ml)*		30 (0-4850)
Operation time (min)*		247 (127-812)
Postoperative complication (Clavien-Dindo) (%)	Grade II	9 (12.0)
	Grade IIIa	12 (16.0)
	Grade ≥ IIIb	0
Postoperative stay (day)*		13 (8-54)
Adjuvant chemotherapy (%)	Yes	37 (71.2**)
Recurrence (%)	Yes	7 (13.5**)

*Median (min-max)

**Percentage with 52 cases of curative resection, including 1 case of peritoneal dissemination

### Open surgery versus laparoscopic surgery

Patient background and operative factors of the 21 patients (28.0%) in the open surgery (open) group and the 54 (72.0%) patients in the laparoscopic surgery (lap) group can be compared in Table 4. There were significantly more T4 cases in the open group. Operation time was 199 min in the open group and 252.5 min in the lap group (*p* = 0.49), and blood loss was 210 mL in the open group and 30 mL in the lap group (*p* = 0.0017). Postoperative complications of Clavien-Dindo classification grade III or higher were observed in 5 patients (23.8%) in the open group and in 7 (13.0%) patients in the lap group. Although anastomotic leakage occurred in 5 patients in the lap group, none of the patients was classified as Clavien-Dindo IIIb or higher.

**Table 4:** Characteristics of the patients, open surgery group (open) versus laparoscopic surgery group (lap)

		Open (*N* = 21)	Lap (*N* = 54)	*p* value**
Sex (%)	M	15 (71.4)	33 (61.1)	0.40
	F	6 (28.6)	21 (38.9)	
Age*		68.0 (50-86)	63.5 (30-82)	0.061
BMI*		19.7 (16.7-28.0)	21.3 (14.4-34.9)	0.73
ASA (%)	1	4 (19.0)	21 (38.9)	0.17
	2-3	17 (81.0)	33 (61.1)	
T (%)	2	0 (0)	1 (1.9)	0.042
	3	8 (38.1)	32 (59.3)	
	4a	8 (38.1)	18 (33.3)	
	4b	5 (23.8)	3 (5.6)	
N (%)	0	10 (47.6)	20 (37.0)	0.37
	1	4 (19.0)	19 (35.2)	
	2	7 (33.3)	15 (27.8)	
Stage (%)	I	0 (0)	1 (1.9)	0.23
	II	8 (38.1)	18 (33.3)	
	III	5 (23.8)	20 (37.0)	
	IV	8 (38.1)	14 (25.9)	
Location (%)	Right	6 (28.6)	10 (18.5)	0.35
	Left	15 (71.4)	44 (81.5)	
Size (mm)*		65.0 (40-108)	61.5 (0-615)	0.36
Operation time (min)*		199 (127-610)	252.5 (137-812)	0.49
Blood loss (ml)*		210 (40-4850)	30 (5-615)	0.0017
Lymph node dissection*		25 (7-72)	25 (8-102)	0.62
Postoperative stay (day)*		14 (8-54)	13 (8-47)	0.60
Postoperative complication, Clavien-Dindo ≥grade III (%)		5 (23.8)	7 (13.0)	0.26
Anastomotic leakage		2	5	
SSI		2	0	
Ileus		1	2	

*BMI* body mass index, *ASA* American Society of Anesthesiologists

*Median (min-max)

**chi-squared test

### OS and RFS

**Fig. 1:**
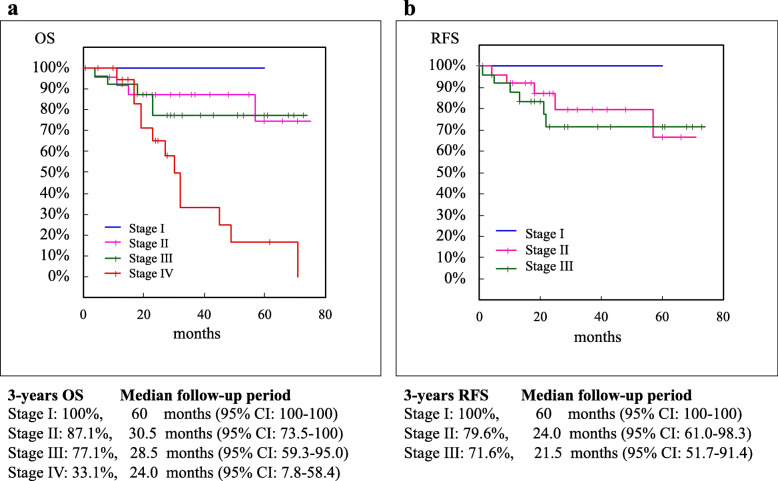
Kaplan–Meier survival curves for patients with MLBO in each stage. **a** OS in Stage I-IV. **b** RFS in stage I-III

**Fig. 2:**
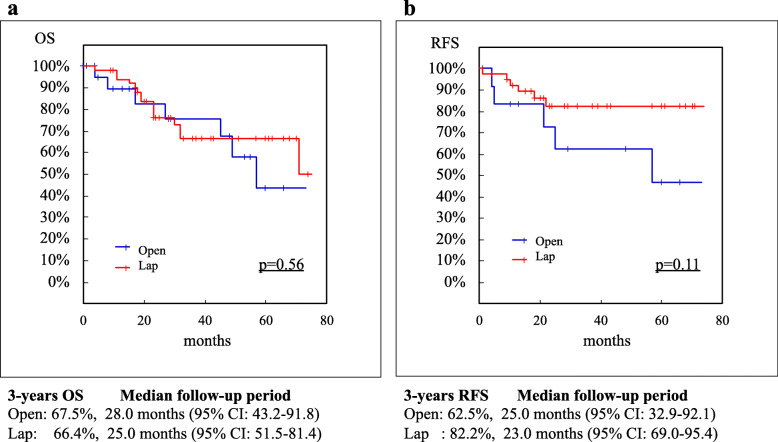
Kaplan–Meier survival curves for patients with MLBO compared by surgical approach. **a** OS in open or laparoscopic surgery group. **b** RFS in open or laparoscopic surgery group

The rate of 3-year OS (median follow-up months) was 100% (60.0) for stage I, 87.1% (30.5) for stage II, 77.1% (28.5) for stage III, and 33.1% (24.0) for stage IV cancer (Fig. 1a). That of 3-year RFS was 100% (60) for stage I, 79.6% (24.0) for stage II, and 71.6% (21.5) for stage III cancer (Fig. 1b). When compared by surgical approach, the 3-year OS rate was 66.4% for the lap group and 67.5% for the open group (*p* = 0.56). The 3-year RFS rate was 82.2% for the lap group and 62.5% for the open group (*p* = 0.11). Although the difference was not statistically significant, the prognosis was relatively better in the lap group (Fig. 2).

## Discussion

Most cases of MLBO are advanced, having a depth of wall invasion deeper than T3, with vascular invasion and positive lymph nodes [7, 21, 22]. There are also some reports that 25% of MLBOs are diagnosed as stage IV, which indicates distant metastases [7]. There is also a report that the long-term prognosis is equivalent to non-obstructed cases when compared by stage [23], but even in the same stage, many cases of MLBO are reported to be advanced cases and to have poorer prognosis [24]. Therefore, patients with stage II MLBO are considered the so-called high-risk group, for which postoperative adjuvant chemotherapy is recommended in the guidelines.

In this retrospective study, we examined the short- and long-term prognoses of MLBO to verify the effectiveness and safety of the BTS strategy using SEMS. Although no direct comparison with emergency surgery was made in this study, no significant difference in either short-term or long-term prognosis of colorectal cancers has been reported in the past. Furthermore, the BTS strategy secures time for systemic examination such as for the presence of multiple cancers. Therefore, we consider SEMS placement to be an effective treatment strategy for MLBO.

**Fig. 3:**
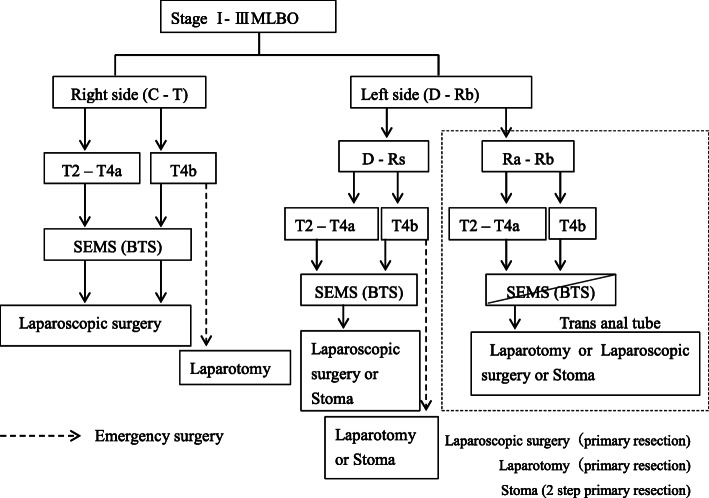
Strategy for MLBO in our institute (TMG (Tajima-Matsuhashi-Gifu University)-BTS category classification)

At present, there is no consensus on the effectiveness of SEMS for BTS in MLBO. The ESGE guidelines emphasize the limitation that SEMS as a BTS should be considered within sufficient skill and expertise [15]. Sensitivity analysis at a meta-analysis study concluded as experience and quantity affect long-term outcomes from the result of a technical success rate of 90% vs. 90% or experience of SEMS cases of < 40 vs. ≥ 40 [25]. This study also revealed that a perforation rate of less than 8% had significantly better 3-year overall survival than studies with a perforation rate of 8% or more. As perforation is reported to be one of the risks of peritoneal dissemination and a poor prognostic factor [26], the success rate of SEMS placement would seem to affect the long-term prognosis. The success rates shown in the cohort studies conducted in Japan were 98–99% with low perforation rates of 0–2%. In the present study as well, only 2 of the 75 patients suffered perforation, and the technical success rate was high at 97.3%, which indicates that SEMS placement could be safely performed. In addition, there is a report that mechanical compression of a tumor with a metallic stent induces perineural invasion and stimulates cancer cells to promote tumor growth and metastasis [27], whereas other reports found no significant difference in perineural invasion compared with a transanal tube. Another report concluded that mechanical compression of the tumor rather decreased the proliferative capacity [28]. Therefore, the effect of metallic stents on long-term prognosis still remains controversial and an important topic. A multicenter randomized controlled trial (COBRA trial) is currently underway in Japan to establish its own evidence. In our institution, SEMS is widely used as a BTS for MLBO on the basis of these results. Figure. 3 shows the strategy in our institution which is described as TMG (Tajima-Matsuhashi-Gifu University)-BTS category classification. The point to be noted in this strategy is that considering emergency surgery including temporary stoma construction rather than SEMS placement in cases of invasion of the other organs (depending on the organs) and in lower rectal cases. However, as shown in the schema, due to reasons of securing the margin for resection and the pain involved after insertion, a transanal ileus tube or emergency surgery is rather recommended for lower rectal cancers (Fig. 3). The indication of the laparoscopic approach is also problematic in advanced cases of MLBO after SEMS placement. While some reports showed the feasibility and safety of laparoscopic surgery after SEMS placement with no transition to open surgery and fewer complications, no reports suggested for long-term prognosis [29, 30]. We also compared laparoscopic surgery and open surgery in this study.

In recent years, the development of surgical devices and endoscopic surgical techniques has led to the widespread use of laparoscopic surgery even for advanced colorectal cancers. However, there is no consensus on a surgical approach for MLBO, especially with colonic stent placement. Law et al. [31] compared short-term treatment after colonic stent placement between open and laparoscopic surgery groups and found that the postoperative hospital stay was shorter and the incidence of postoperative complications was lower in the laparoscopic surgery group. Other reports on BTS also showed that laparoscopic surgery tended to be performed on patients in whom effective decompression was achieved [32]. In the early stage of BTS, we performed the operation by laparotomy, but laparoscopic surgery has gradually increased over time and is presently the first choice. However, we still perform laparotomy in patients with T4b cancer.

Although the incidence of anastomotic leakage was somewhat high in the Lap group in the present study, there was no complication higher than Clavien-Dindo grade 3b that required reoperation. For this reason, left-sided colorectal cases (including RS, Ra, Rb) tended to be included in the lap group.

Regarding long-term prognosis in the study patients, 3-year OS was over 80% for all stages, and there was no significant difference between open and laparoscopic surgery. These results suggest that the combination of SEMS placement with laparoscopic surgery may be a feasible and safe treatment.

In patients with colorectal cancer, the prevalence of synchronous cancers ranges from 0.7 to about 7% [33, 34]. However, synchronous multiple cancers were detected in 14.7% of patients in our institution. Therefore, we perform total colonoscopy including the oral side of the primary tumor in our institution before performing BTS to avoid an unnecessary second colorectal resection.

As the limitations, this is a retrospective, single-arm study and should better to be compared by factors such as tumor localization and postoperative treatment which may affect RFS and OS. Moreover, since this study does not compare the primary anastomosis case with the stoma construction case, it is difficult to concluded that the stoma construction can be safely avoided.

## Conclusion

In this study, preoperative SEMS treatment has a potential for avoiding the construction of a stoma and allows for anastomosis. Moreover, even when multiple cancers are found by preoperative examination after SEMS placement, the ability to perform one-time surgery safely contributes to the minimally invasive treatment. The short- and long-term results for preoperative SEMS placement for MLBO were considered to be acceptable. We recommend total colonoscopy before performing BTS because of the tendency for synchronous multiple cancers to exist with higher probability.

## Supplementary information

**Additional file 1: Table S1.** Details of each postoperative complication cases with more than Clavien-Dindo grade IIIa.

## Data Availability

Not applicable

## Abbreviations

ASGE: American Society of Gastrointestinal Endoscopy
BTS: Bridge to surgery
CROSS: ColoRectal Obstruction Scoring System
CT: Computed tomography
ESGE: European Society of Gastrointestinal Endoscopy
MLBO: Malignant large-bowel obstruction
OS: Overall survival
RFS: Relapse­free survival
SEMS: Self-expandable metal stent
UICC: Union for International Cancer Control
