# Supplementary material for: Short- and long-term outcomes after colonic self-expandable metal stent placement for malignant large-bowel obstruction as a bridge to surgery focus on the feasibility of the laparoscopic approach: a retrospective, single center study
Source: World J Surg Oncol. 2020 Oct 13;18:265. doi: 10.1186/s12957-020-02039-8 (PMC7556958; doi:10.1186/s12957-020-02039-8)
Supplement: Supplementary file 1 — Additional file 1: Table S1. Details of each postoperative complication cases with more than Clavien-Dindo grade IIIa. [file 12957_2020_2039_MOESM1_ESM.docx]

**Supplemental table**

| No. | Age | Sex | Interval to surgery | Tumor location | Synchronous cancers | Surgery | | | Complication | hospital stay (day) | Stage | Recurrence | outcome |
| --- | --- | --- | --- | --- | --- | --- | --- | --- | --- | --- | --- | --- | --- |
|  |  |  |  |  |  | Approach | Blood loss (ml) | Time (min) |  |  |  |  |  |
| 1 | 61 | F | 11 | S | No | Lap | 113 | 253 | Anastomotic leakage | 34 | IIB | Yes | alive |
| 2 | 68 | M | 10 | Ra | No | Open | 210 | 197 | SSI | 25 | IVA | - | dead |
| 3 | 36 | F | 7 | RS | No | Lap | 10 | 157 | Anastomotic leakage | 32 | I | - | alive |
| 4 | 70 | M | 7 | S | No | Lap | 5 | 176 | Anastomotic leakage | 31 | IIB | - | alive |
| 5 | 48 | M | 16 | RS | No | Lap | 150 | 428 | Ileus | 25 | IVC | - | alive |
| 6 | 71 | M | 34 | RS | No | Open | 220 | 199 | Ileus | 18 | IVA | - | dead |
| 7 | 59 | M | 12 | S | No | Open | 990 | 408 | Anastomotic leakage | 43 | IIIC | Yes | alive |
| 8 | 73 | F | 18 | S | No | Open | 4850 | 610 | SSI | 32 | IIIC | Yes | dead |
| 9 | 66 | M | 13 | T | No | Open | 950 | 302 | Anastomotic leakage | 54 | ⅡC | - | alive |
| 10 | 44 | M | 8 | RS | No | Lap | 50 | 346 | Anastomotic leakage | 47 | IVA | - | dead |
| 11 | 64 | M | 25 | S | Yes (T) | Lap | 20 | 247 | Anastomotic leakage | 42 | ⅡA | - | alive |
| 12 | 73 | M | 24 | D | Yes (S) | Lap | 5 | 252 | Ileus | 27 | ⅢC | Yes | dead |

F: Female, M: Male, T: Transverse colon, D: Descending colon, S: Sigmoid colon, RS: Rectosigmoid colon, Ra: Rectum above the peritoneal reflection,

SSI: Surgical Site Infection

F: Female, M: Male, T: Transverse colon, D: Descending colon, S: Sigmoid colon, RS: Rectosigmoid colon, Ra: Rectum above the peritoneal reflection, SSI: Surgical Site Infection
